# Aquatic macrophytes and macroinvertebrate predators affect densities of snail hosts and local production of schistosome cercariae that cause human schistosomiasis

**DOI:** 10.1371/journal.pntd.0008417

**Published:** 2020-07-06

**Authors:** Christopher J. E. Haggerty, Sidy Bakhoum, David J. Civitello, Giulio A. De Leo, Nicolas Jouanard, Raphael A. Ndione, Justin V. Remais, Gilles Riveau, Simon Senghor, Susanne H. Sokolow, Souleymane Sow, Caitlin Wolfe, Chelsea L. Wood, Isabel Jones, Andrew J. Chamberlin, Jason R. Rohr

**Affiliations:** 1 Department of Biological Sciences, Environmental Change Initiative, Eck Institute of Global Health, University of Notre Dame, Notre Dame, Indiana, United States of America; 2 Department of Integrative Biology, University of South Florida, Tampa, Florida, United States of America; 3 Université Cheikh Anta Diop, Dakar, Senegal; 4 Department of Biology, Emory University, Atlanta, Georgia, United States of America; 5 Department of Biology, Hopkins Marine Station, Stanford University, Pacific Grove, California, United States of America; 6 Station d'Innovation Aquacole, Saint-Louis, Senegal; 7 Centre de Recherche Biomédicale Espoir pour la Santé, Saint-Louis, Senegal; 8 Division of Environmental Health Sciences, School of Public Health, University of California, Berkeley, California, United States of America; 9 Institut Pasteur de Lille—CIIL, France; 10 Woods Institute for the Environment, Stanford University, Stanford, California, United States of America; 11 College of Public Health, University of South Florida, Tampa, Florida, United States of America; 12 School of Aquatic and Fishery Sciences, University of Washington, Seattle, Washington, United States of America; University of Oxford, UNITED KINGDOM

## Abstract

**Background:**

Schistosomiasis is responsible for the second highest burden of disease among neglected tropical diseases globally, with over 90 percent of cases occurring in African regions where drugs to treat the disease are only sporadically available. Additionally, human re-infection after treatment can be a problem where there are high numbers of infected snails in the environment. Recent experiments indicate that aquatic factors, including plants, nutrients, or predators, can influence snail abundance and parasite production within infected snails, both components of human risk. This study investigated how snail host abundance and release of cercariae (the free swimming stage infective to humans) varies at water access sites in an endemic region in Senegal, a setting where human schistosomiasis prevalence is among the highest globally.

**Methods/Principal findings:**

We collected snail intermediate hosts at 15 random points stratified by three habitat types at 36 water access sites, and counted cercarial production by each snail after transfer to the laboratory on the same day. We found that aquatic vegetation was positively associated with per-capita cercarial release by snails, probably because macrophytes harbor periphyton resources that snails feed upon, and well-fed snails tend to produce more parasites. In contrast, the abundance of aquatic macroinvertebrate snail predators was negatively associated with per-capita cercarial release by snails, probably because of several potential sublethal effects on snails or snail infection, despite a positive association between snail predators and total snail numbers at a site, possibly due to shared habitat usage or prey tracking by the predators. Thus, complex bottom-up and top-down ecological effects in this region plausibly influence the snail shedding rate and thus, total local density of schistosome cercariae.

**Conclusions/Significance:**

Our study suggests that aquatic macrophytes and snail predators can influence per-capita cercarial production and total abundance of snails. Thus, snail control efforts might benefit by targeting specific snail habitats where parasite production is greatest. In conclusion, a better understanding of top-down and bottom-up ecological factors that regulate densities of cercarial release by snails, rather than solely snail densities or snail infection prevalence, might facilitate improved schistosomiasis control.

## Author summary

Over 800 million people are at risk of schistosomiasis and environmental factors that regulate densities of cercariae parasites that infect humans remain poorly understood. We sampled a spatially extensive area at 36 water-access points in northern Senegal, and quantified densities of snail intermediate hosts, snail predators, and aquatic vegetation in each sample, as well as cercariae released from snails after they were brought to the laboratory. We found that the quantity of submerged aquatic vegetation, particularly *Ceratophyllum* spp., was positively associated with schistosome cercariae released per infected snail, and total potential cercariae released by the collected snails per water access site. In contrast, the abundance of aquatic predators near infected snails (in the same sweep) was negatively associated with the per-capita cercarial release by infected snails, but positively associated with total snail abundance per site. Additionally, snail densities and potential cercarial densities (estimated as the sum of cercariae released by all collected, infected snails at a site) were only weakly correlated, suggesting that snail densities alone might not accurately reflect total potential of those snails to emit schistosome cercariae. Overall, a better understanding of aquatic factors that can influence the production of schistosome cercariae under field conditions, rather than snail host abundance alone, might facilitate improvements in schistosomiasis monitoring and control.

## Introduction

Schistosomiasis is caused by a snail-transmitted trematode that affects >200 million people in 76 countries, with >800 million at risk [1–5]. The disease is among the top three highest burden infections among neglected tropical diseases, and disproportionally affects children [1, 5]. Humans are infected when their skin is penetrated by cercariae—the free-swimming larval stage of schistosomes—which are released into freshwaters by infected intermediate host snails. The cercariae develop into adult worms in veins near the human gastrointestinal or urinary tract (*Schistosoma mansoni* and *S*. *haematobium*, respectively). There, they produce eggs that pass through the intestine or bladder and are released with feces or urine, respectively, into the water to hatch into miracidia and infect new snails [6]. Infection in humans can result in a loss of tissue function, stunted growth, learning deficits, and in some cases, increased risk of HIV, infertility, bladder cancer, liver failure, and death [7–9]. While relatively inexpensive drugs exist to clear infections, such as praziquantel, these drugs are not available to the majority of at-risk populations in Africa and offer no protection against reinfection, and humans are often re-exposed when they return to waterbodies with snails releasing schistosome cercariae [10, 11].

While snail control has played an important role in reducing schistosomiasis prevalence in several countries [12], many studies have failed to demonstrate an association between the abundance of the snail hosts and prevalence in humans at a site [13–15] (but see[16]). A major limitation of this prior work has been the focus on total snail abundance or prevalence, without considering per-capita cercarial release by snails. In other words, high snail densities have not been reliably associated with high prevalence in humans [15], even at water access sites with high human contact [17]. Snail control efforts that make considerable reductions in total snail numbers at water-access points can sometimes fail if infected snails remain [18], and cercarial concentrations measured in natural waters do not always correlate with infected snail numbers [19]. Importantly, recent work in aquatic mesocosms has further demonstrated that snail abundances do not always correspond reliably with cercarial abundances [20]; therefore, factors influencing cercarial production by infected snails could be better predictors of human disease risk than snail population size alone. Past work has found that some water quality parameters can influence miracidial and cercarial viability [21, 22], but the ecological factors influencing cercarial release from snails, a valuable measure of human exposure, has not been well investigated. Finally, snails occur heterogeneously within their habitat and across seasons or years [16, 23], meaning that both infected snail and cercarial densities are highly variable [24, 25]. This can further complicate attempts to correlate snail or cercarial abundance to human cases of schistosomiasis.

Recent experimental work has demonstrated that aquatic environmental factors affecting snail density and snail food resources (periphyton growing on surfaces) can influence cercarial production per snail and perhaps ultimately human exposure risk, but these patterns had not been previously validated by field studies [20]. Aquatic vegetation provides some of the highest surface-area habitat for snails upon which periphyton (attached algae and biofilms) can grow [26]. Thus, submerged aquatic vegetation could have bottom-up (i.e. resource dependent) ecological effects that might impact population growth of snails and cercarial production per infected snail [20, 27, 28]. Conversely, macroinvertebrate predators of snails can induce anti-predator behavior that reduces snail foraging activity, growth, and density [26, 27, 29, 30], and many macroinvertebrates are also important predators of trematode cercariae [31]. Thus, macroinvertebrates could have top-down effects that might be lethal and/or sublethal to both snails and cercariae. Sublethal effects could involve predators suppressing snail feeding because of fear effects and anti-predator escape or hiding behavior [32]. Reduced feeding could lead to lower rates of individual growth and lower energy available for cercarial production in infected snails [33, 34]. Additionally, recent work has found that agrochemicals, particularly fertilizers, pesticides, and herbicides, could fuel schistosomiasis transmission by increasing aquatic resources for snails or by killing snail predators [26].

**Fig 1:**
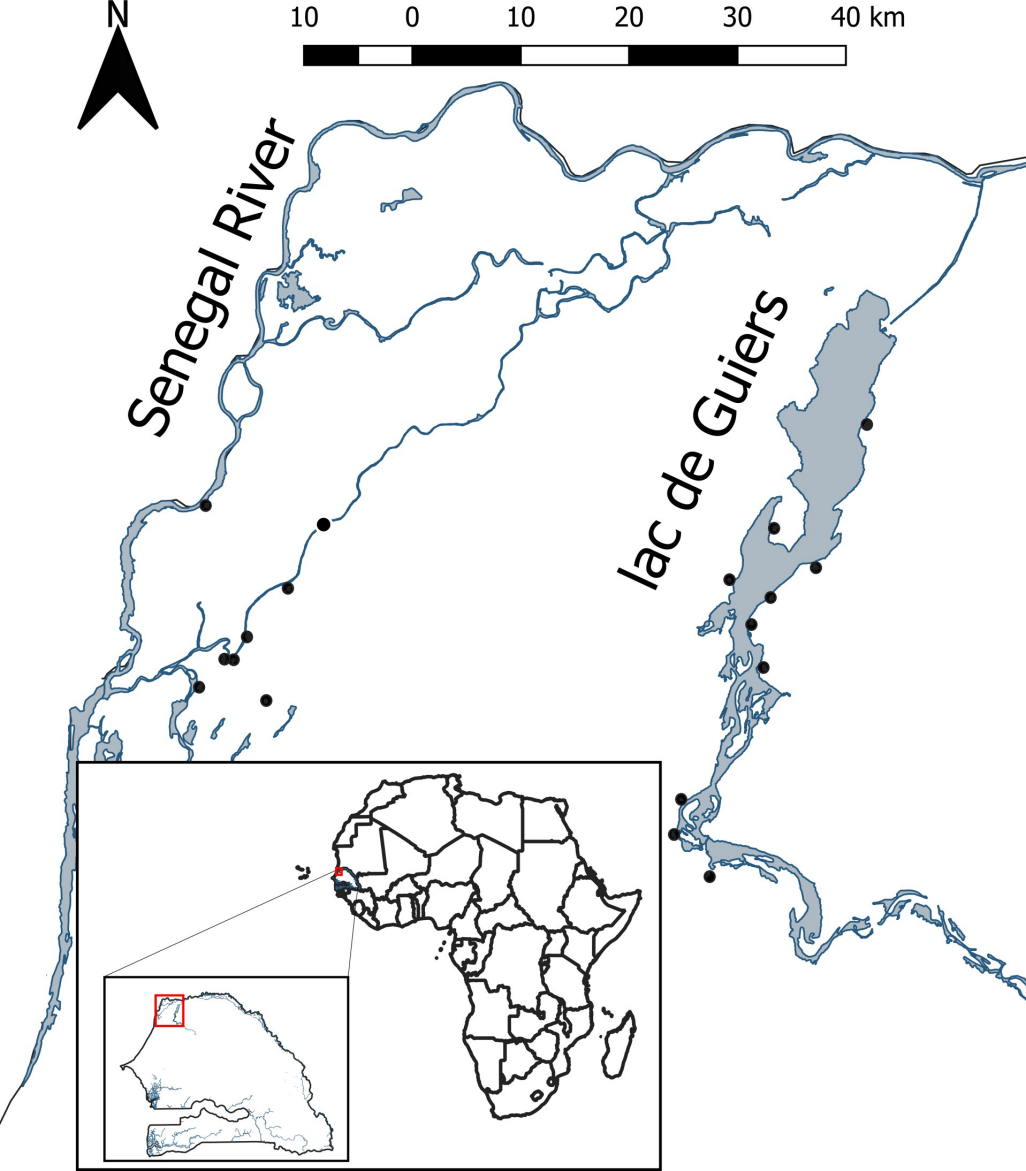
Map of 18 sampled villages located along the lower Senegal River (left village cluster) and “Lac de Guiers” regions (right village cluster) of northern Senegal. All village point locations are displayed on a single map enlarged from red polygon of the inset map on the lower left showing the country of Senegal within the African continent. Fig 1 was created using the Africa Basemap in ESRI 2011. ArcGIS Desktop: Release 10. Redlands, CA: Environmental Systems Research Institute. Waterbodies within Senegal were selected using the Land cover of Senegal—Globe cover Regional dataset available from the Food and Agriculture Organization of the United Nations (http://www.fao.org/geonetwork/srv/en/main.home?uuid=ba4526fd-cdbf-4028-a1bd-5a559c4bff38).

The objectives of our study were to investigate whether aquatic vegetation and/or macroinvertebrate predators influenced 1) the density or size of snail hosts or 2) per-capita cercarial production of schistosome-transmitting snails or 3) snail infection prevalence. To accomplish this, we sampled July–August 2018 when air temperature seasonally peaks, increasing human use of water access sites; this time period represents a key transmission period for schistosomiasis in Senegal, West Africa [20]. We quantified snails, vegetation, and macroinvertebrates in each of 15 1-m aquatic dip net sweeps at 36 water-access points across 18 villages in the lower basin of the Senegal River (Fig 1). We quantified the number of schistosome cercariae released from each collected snail in the laboratory on the day of its collection. We hypothesized that the abundance of aquatic vegetation would be positively associated with the abundance of snails known to harbor human schistosomes, per-capita cercarial release by snails, and the sum of cercariae across all snails encountered in sweeps at a water access site. We also hypothesized that macroinvertebrate predators would be correlated with snails and cercarial abundance, but we did not have an *a priori* hypothesized direction for either relationship, because predators could share habitat with or track snails in space, creating a positive correlation, or they could reduce snail densities through predation, driving a negative correlation. Additionally, based on the mechanisms we outline above, we hypothesized that macroinvertebrate predators would be negatively associated with the per-capita cercarial production by snails and the total potential cercariae released by snails at a site. To our knowledge, these effects have not been quantified previously (but see [34]). Again, we did not have an *a priori* hypothesized direction for between predator abundance and cercarial abundance because the relationship may depend on whether the effect of snail predators is predominantly lethal or sublethal. If these small macroinvertebrate predators have a strong lethal effect on snails, the surviving snails could be released from competition, thus increasing per-capita resources, snail growth rate, and cercarial production per infected snail [20]. If predators have a predominantly sublethal effect on snails, then reduced per-capita foraging and resource intake rates could lower cercarial production [32–34]. The sum of cercariae released from all snails encountered at a site was expected to be influenced by the complex interplay of the total snail abundance, snail size distribution, snail infection prevalence, and each snail’s individual physiologic status (and thus per-capita cercarial production; i.e., either good body condition and available energy leading to high per-capita shedding rates, or energy stress leading to lower per-capita shedding rates), with energy stress caused by low food availability, fear and anti-predatory behaviors, or a combination of these effects. We also investigated whether snail infection prevalence was dependent upon the abundance of aquatic vegetation or macroinvertebrate predators of snails, but we did not have directional hypotheses for these relationships as we did for the effects on snail abundance, snail size, or per-capita cercarial production, because it was not clear to us *a priori* how aquatic vegetation and insect predators would affect snail infection prevalence given that it might be more likely to vary with human deposition of schistosome eggs at a site than these other response variables [35]. Similarly, although crustacean predators of snails, such as *Macrobrachium* spp., preferentially consume infected snails [32], which might reduce prevalence, these predators were at low abundance in our study sites and it is unclear whether small macroinvertebrate predators we sampled would exhibit the same preference for infected snails.

## Methods

### Sampling protocols

We surveyed snails between July and August of 2018 at 18 villages located along the lower Senegal River and along the shore of Lac de Guiers (16°15′N 15°50′W), a lake south of the city of Richard Toll (Fig 1). At each village, there are between one and four human water-access points (36 water-access points were surveyed in total), typically bordered by dense emergent vegetation, such as *Typha* or *Phragmites* spp. We created sampling polygons at each access point that included all open water and floating vegetation and extended laterally several meters into the emergent vegetation border. The outer limit of each sampling polygon ran from the shore to the outer limit of emergent vegetation, which often encompassed water depths greater than those where dip net sampling is possible. We created random sampling points within each polygon and at each performed a 1-m net sweep using a 40.6 x 45.7 cm D-frame dip net, with 60.7 cm depth and 3mm mesh, to encounter all aquatic vegetation and the sediment surface. We stratified 15 points per site across three potential snail habitats (open water, *Ceratophyllum*, or other vegetation) based on their proportional cover within each polygon.

The cover of each microhabitat was determined using an aerial photograph taken between January 10 and January 23 2018 and verified visually by technicians on the shore of the water-access point prior to sampling. After three sweeps in one microhabitat with zero snail captures, we allocated the remaining points to other microhabitats based on their cover. We located points in the field using a Trimble R1 GPS unit to within ± 1 m accuracy and took net contents to the shore, where we collected all snails, counted and identified macroinvertebrate predators to taxonomic order using [36], and weighed the mass of submerged plant, *Ceratophyllum* spp., captured by the sweep net. We weighed *Ceratophyllum* spp. because it was by far the most abundant type of submerged vegetation at most sites. We recorded the presence of other aquatic vegetation encountered in our sweep nets (e.g., *Ludwigia* spp. or *Cyperus* spp.).

We elected to take field collected snails to the lab and individually shed them in the afternoon on their day of capture using standardized conditions because *S*. *mansoni* and *S*. *haematobium* cercariae that infect humans are generally shed in the afternoon from 11:00 to 16:00, whereas non-human schistosomes such as *S*. *bovis* (also common in this region) are generally shed in the morning from 07:00 to 10:00 [37]. Hence, shedding in the afternoon is more likely to capture human schistosomes and thus not confound human and cattle schistosomes. We report potential locally produced cercarial densities by encountered snails at a sweep or site by using per-capita cercarial release and summing cercarial counts from all infected snails at a site, respectively. We present analyses using snail counts per sweep (sweep-level) and by summing counts from all sweeps at a water-access point (site-level).

We targeted our snail field collection between 11:00 and 14:00 hours because it corresponds to the time of day when non-human schistosome species such as *S*. *bovis* have past their peak emergence and have stopped shedding (see above). Snails in each sweep were placed into a clear vial and transported within 2 hours to the laboratory. At each water access site, we recorded water temperature 4cm below the water surface using a YSI Pro Plus and 5560 conductivity/temperature probe. In the laboratory, snails from each sweep were identified as *Bulinus* spp. or *Biomphalaria pfeifferi*, which are the local snail intermediate hosts to human schistosomes. *Bulinus* spp. or *B*. *pfeifferi* snails were counted, photographed, and then placed into individual 3.5 mL wells filled with distilled water at approximately 25° C. These welled plates were placed under a fluorescent lamp as a standard method used to encourage cercarial emergence within 1 hr [38, 39] during the afternoon, between 16:00 and 19:00 hours, which overlaps the peak daily cercarial emergence period for human schistosomes *S*. *haematobium* and *S*. *mansoni* [37]. We chose to shed for only 1 hr because it *i*) maximized the overlap in the peak emergence period of human schistosomes in our study, *ii*) minimized overlap with other time periods where non-human schistosomes are often shed and *iii*) is not uncommon for >1,000 cercariae to be shed within a 1-hr period in the lab [40]. Thus, we could focus our shedding period to one hour because we targeted the emergence period of human schistosomes [37], and shedding for longer periods i.e. 24 hrs might increase overlapping peak emergence of cryptic and indistinguishable cercariae in our study region that are non-human-infecting (e.g. *S*. *bovis*). After one hour, snails were removed from the vials and Lugol’s solution was added to stain and kill the cercariae. The diameter of each snail shell was later quantified from the photographs using ImageJ software (https://www.imagej.nih.gov/ij/). Human schistosome cercariae were counted in each well and differentiated from other fork-tailed cercariae under the microscope using morphological features including fork length of the tail, eyespots, and finfolds [41, 42].

### Data analyses

All statistical analyses were conducted in *R* version *3*.*4*.*2* (https://www.r-project.org/). Despite the large number of snail captures in our study, snail infection prevalence was low, limiting our sample size for cercarial analyses. We attempted to deal with this problem using a hierarchical regression approach to include as many data points and thus retain as much power as possible at each step in our analyses. More specifically, we used data from all sweeps (*n* = 540) for snail abundance or snail size analyses (S1–S3, S6 Tables), and data only from sweeps with infected snails (*n* = 39) for analyses of cercarial release per snail or site (S4 and S5 Tables). We then attempted to combine all analyses using a piecewise path model that examined both direct and indirect relationships. However, we could not find a method that allowed us to account for the hierarchical nature of our dataset described above. Thus, predictors and responses had to be averaged at the site-level, which resulted in a loss of statistical power compared to our regression analyses (S1 Table). We did not control for spatial autocorrelation because a previous study at the same 16 of 18 villages used in this analysis revealed that spatial terms were non-significant for snails [16].

### Effects of aquatic vegetation and predators on snail hosts

**Fig 2:**
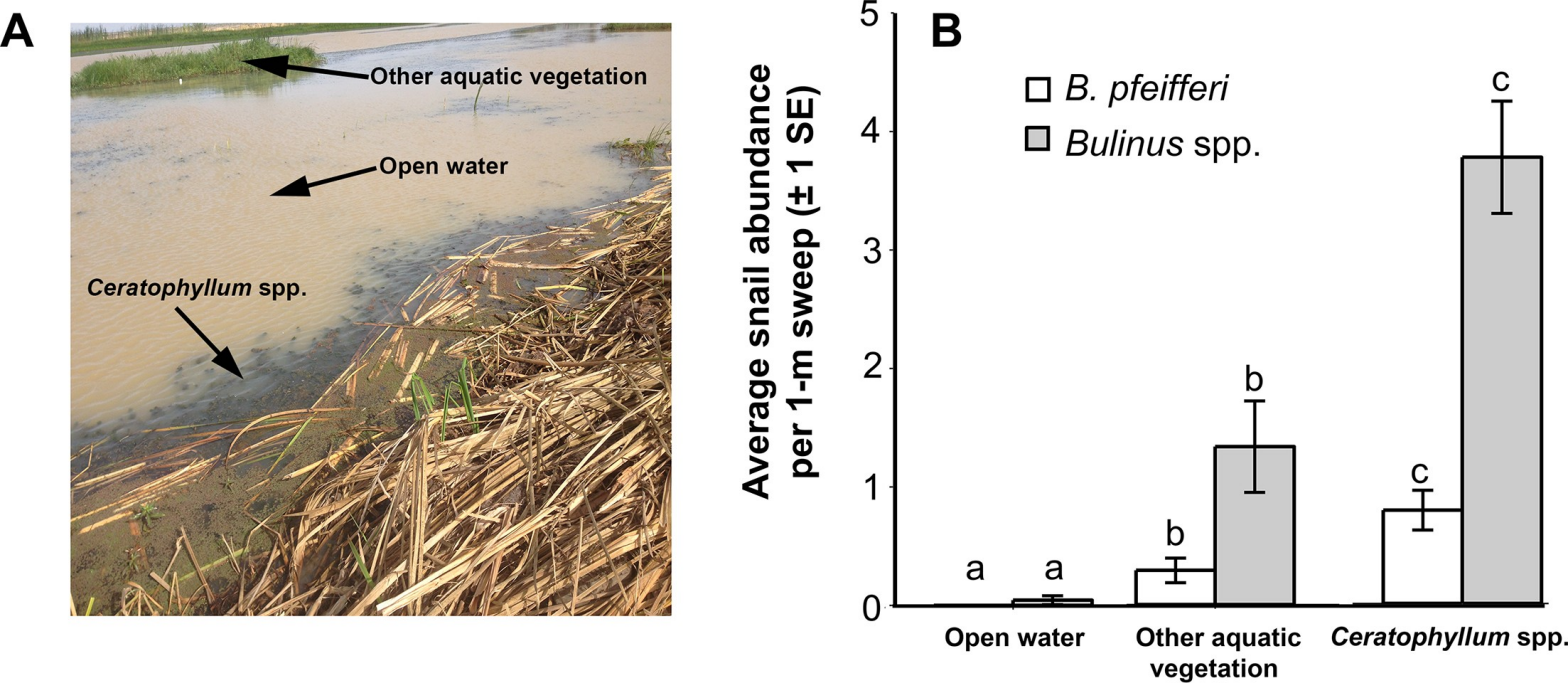
**Photograph of different aquatic microhabitats we sampled (A) and average snail abundance per 1-m sweep found in three microhabitats: open water, aquatic vegetation other than *Ceratophyllum* spp. (typically *Ludwigia*, *Cyperus*, and *Typha* spp.), and *Ceratophyllum* spp. (B).** Symbols above error bars (± 1 SE) denote significantly different snail abundance among aquatic microhabitats using separate Tukey's post-hoc comparisons for *Bulinus* and *Biomphalaria*.

To compare snail abundance across aquatic microhabitats, we used negative binomial regression in the R package *lme4* (*glmer*.*nb* function) with snail counts per sweep as the dependent variable, microhabitat type (open water, *Ceratophyllum*, or other vegetation; Fig 2) as the independent variable, and water-access point nested within village as a random effect. We then used a Tukey's post-hoc multiple comparison test (using the *lsmeans* package) to determine which microhabitats were significantly different from one another.

To determine whether snail abundance in a sweep was related to aquatic vegetation and macroinvertebrates in the same sweep, we performed separate negative binomial models with either *Bulinus* spp. or *Bi*. *pfeifferi* counts as the response variable, vegetation mass and total abundance of macroinvertebrate predators as independent variables, and water-access point nested within village as a random effect. We then conducted a negative binomial model using the same fixed effects as just described, but we changed our response variable to the site-level total snail counts and included a random effect of village. This model allowed us to evaluate whether the sweep-level patterns were consistent at the site level. To examine the relative influence of individual predator taxa, we also performed the same sweep-level regression described above but replaced total invertebrates with the counts of invertebrates in each taxon. Taxon-level analyses were consistent with total invertebrate trends for both *Bulinus* and *Biomphalaria*, and thus, taxon-level results are presented in the supplementary material. We unfortunately did not have sufficient data on the abundance of each taxon within sweeps having infected snails (due to low snail infection) to model the influence of individual predator taxa on cercarial production.

To determine how average snail size (mm) varied with vegetation and predators at the sweep-level, we performed separate linear mixed-effects models using the lme function of the *nlme* package (Gaussian-error distribution), with number of intermediate-host snails, *Ceratophyllum* mass (g), and total invertebrate predators as fixed predictors, and water-access point nested within village as a random effect. We then conducted a linear model using the same fixed effects as just described, but we changed our response variable to the site-level average snail size and included a random effect of village.

### Effects of aquatic vegetation and predators on cercarial abundance and snail infection

To assess how ecological factors affected cercarial outputs, we performed a negative binomial regression with per-capita cercarial release by infected snails as the response variable, with one individual-level predictor, shedding snail size (mm), and four sweep-level predictors: snail abundance, macroinvertebrate predator abundance, mass of *Ceratophyllum* (g), and average snail size (longest shell length in mm) per sweep with infected snails (to examine competitive effects), and a random effect of sweep nested within site within village. We did not include a factor for infected snail species because we found it to be not significant in any model that included *Ceratophyllum* mass; thus, we pooled parasite production across snail species. We then conducted a negative binomial regression with site-level potential cercarial production (estimated as the sum of cercariae released by all infected snails found at a site) as the response variable using the same predictors as just described, but including snail infection prevalence at a site or sweep. We did not include a random effect in the analysis of potential site-level cercarial production because it did not converge when random effects were included, likely because of a low number of sites with infected and shedding snails (*n* = 11) relative to the number of predictors (*n* = 6). To model *Bulinus* spp. infection prevalence, we used a binomial mixed effects model using the same sweep-level predictors as described above, with a random term for site nested within village. Due to sample size limitations and low variability across sites, we were unable to model *Bulinus* spp. infection prevalence at the site-level because it was typically below one percent.

For all statistical models, a model selection approach was employed starting with a full model (S2–S6 Tables) and comparing nested models (i.e., one, two, or no predictors and an intercept-only model) using Akaike Information Criteria (AIC), by sequentially dropping single predictors having the lowest ΔAIC from the top model until all ΔAIC were ≥ 2. The significance of parameter estimates was then determined with log-likelihood ratio tests using the *car* package. After model selection, we used the *visreg* package to plot the partial effects of vegetation mass or predator abundance on the response variable while controlling for all other factors in the statistical models (i.e., partial residual plots). To determine whether infected and total snail abundances were associated at the water-access point (site-level), we also performed a Spearman's rank order correlation. We did not include interactions between predictors in our statistical models to limit our type II error rate and because the low number of sites with infected snails (*n* = 11) led to convergence problems as models became more complex.

### Site-level piecewise-path model

Path modeling combines several linear regression models to examine multivariate relationships and thus estimates the relative strengths of all hypothesized direct and indirect causal pathways among variables simultaneously. We used a piecewise-path model in the *piecewiseSEM* package in R [43], because it allowed us to piece together many of the separate generalized linear regressions considered previously, while it is less vulnerable to small sample size limitations than traditional path analysis, and it allowed us to have a mix of error distributions. However, our path model was limited to predictors averaged across all sweeps at a site (S1 Table), and thus is presented in the supplement because our hierarchical regression approach retains the greatest statistical power (see Intro). Furthermore, we only considered site-level prevalence via piecewise SEM, and present model results with and without snail infection prevalence because we think that extremely low snail infection prevalence likely inflated the rate of false negatives. For path model selection, we began with a full model (S1 Table), used tests of d-separation to examine potential missing pathways, individually dropped non-significant paths, and compared nested models using an AIC criterion.

## Results

We performed 540 net sweeps (*n* = 271, 178, and 91 for open water, *Ceratophyllum*, and other vegetation microhabitats, respectively) and sampled approximately 222 kg of vegetation, capturing 1,230 *Bulinus* spp. and 258 *B*. *pfeifferi* snails. Average water temperature was 29.1° C ± 0.2), and *B*. *pfeifferi* snails were found at 67 percent of villages (12/18), with *Bulinus* spp. present at all 18 villages. We found a strong positive Spearman's rank correlation between total schistosome intermediate-host snails and infected snails at the site level (*S* = 65.1, *rho* = 0.70, *p* = 0.016).

### Effect of aquatic vegetation and predators on snail hosts

The number of schistosome intermediate-host snails varied by aquatic microhabitat (*χ*^*2*^ = 61.1, *df* = 2, *p* < 0.001), and Tukey's post-hoc comparisons were significant for all pairwise comparisons among the three snail habitats (Fig 2A; *p* ≤ 0.002). Snails were found at the highest densities in the common submerged macrophyte *Ceratophyllum* spp., which was often mixed with other floating and rooted submerged vegetation; the next highest densities were found in emergent aquatic vegetation, and at the lowest densities were found in open water (S2 Table; Fig 2B).

**Fig 3:**
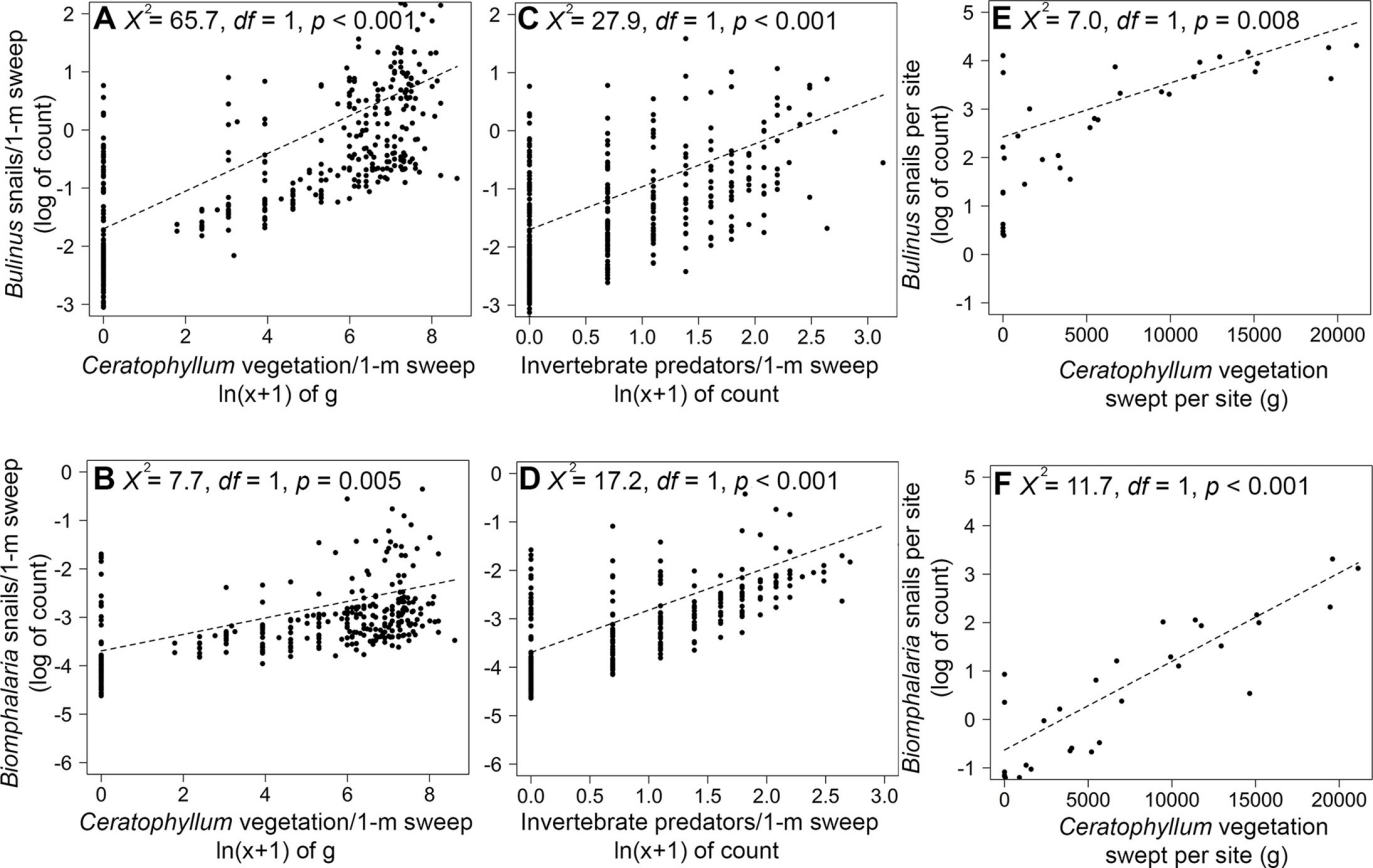
**Partial residual plots based on a negative binomial multiple regression counts of *Bulinus* spp. snails at the sweep (A and C; *n* = 540) or site level (E; *n* = 36) and *Biomphalaria pfeifferi* snails at the sweep (B and D; *n* = 540) or site level (F; *n* = 36) level as the dependent response variables and *Ceratophyllum* spp. mass and invertebrate predator abundance as independent variables.** Plots were generated using *visreg* and show the effect of one factor while controlling for the other. The effects of *Ceratophyllum* spp. mass on snail counts were consistent across sweep and site levels, whereas invertebrate effects were not observed at the site level.

**Fig 4:**
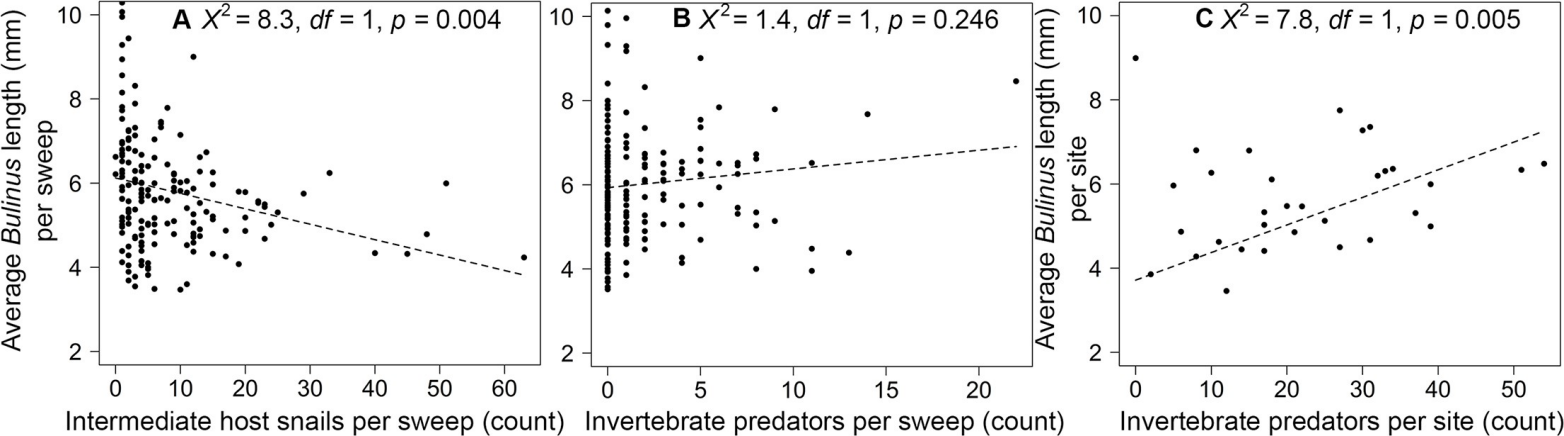
**Partial residual plots from a linear mixed effects regression at the sweep-level showing the effect of abundances of intermediate host snails (A) and invertebrate predators (B) on average *Bulinus* spp. snail length.** Partial residual plots from a linear mixed effects regression at the site-level (*n* = 36) showing the effect of invertebrate predator abundance (C) on average *Bulinus* snail length. Invertebrate predator effects on snail size were positive at both the site level and sweep level, but only significant at the site level.

For all statistical models at the sweep level, ΔAIC values favored both aquatic vegetation mass and invertebrate abundance as predictors for abundance of both snail species (S3 Table). At the sweep level, *Ceratophyllum* spp. mass was positively associated with the number of both *Bulinus* (*χ*^*2*^ = 65.7, *df* = 1, *p* < 0.001; Fig 3A) and *B*. *pfeifferi* snails (*χ*^*2*^ = 7.7, *df* = 1, *p* = 0.005; Fig 3B). These patterns were also supported at the site level (water access site; S4 Table) for *Bulinus* (*χ*^*2*^ = 7.0, *df* = 1, *p* = 0.008; Fig 3E) and *B*. *pfeifferi* (*χ*^*2*^ = 11.7, *df* = 1, *p* < 0.001; Fig 3F). *Ceratophyllum* spp. was present at 75% of water-access points we sampled. Schistosome intermediate hosts were found at only 3 of 9 water-access points lacking *Ceratophyllum* spp. Average *Bulinus* spp. shell diameter per sweep (5.4 mm ± 0.06) was negatively related to snail abundance (*χ*^*2*^ = 8.3, *df* = 1, *p* = 0.004; Fig 4A), but at the site level, *Ceratophyllum* spp. mass (*p* = 0.783) and snail abundance (*p* = 0.927) were not significant predictors of average *Bulinus* spp. snail shell diameter (S5 Table). *B*. *pfeifferi* average shell diameter (8.1 mm ± 0.15) was not significantly associated with any aquatic factors (*p* > 0.05), probably because of the lower captures of, and thus lower statistical power for, this species. *Bulinus* spp. infection prevalence per sweep was positively associated with average snail shell diameter (*χ*^*2*^ *=* 5.4, *df =* 1, *p =* 0.020; S1A Fig) and negatively related to snail abundance (*χ*^*2*^ *=* 5.1, *df =* 1, *p =* 0.023; S1B Fig), but not related to *Ceratophyllum* (*p* = 0.884). Piecewise SEM modeling also found that *Ceratophyllum* mass was the strongest positive predictor of snail abundance (S2 and S3 Figs), and also a positive predictor of snail prevalence at the site level. In summary, *Ceratophyllum* spp. mass was associated with increased snail abundance, with effects consistent across both sweep and site scales, and was positively associated with snail prevalence at the site level.

**Fig 5:**
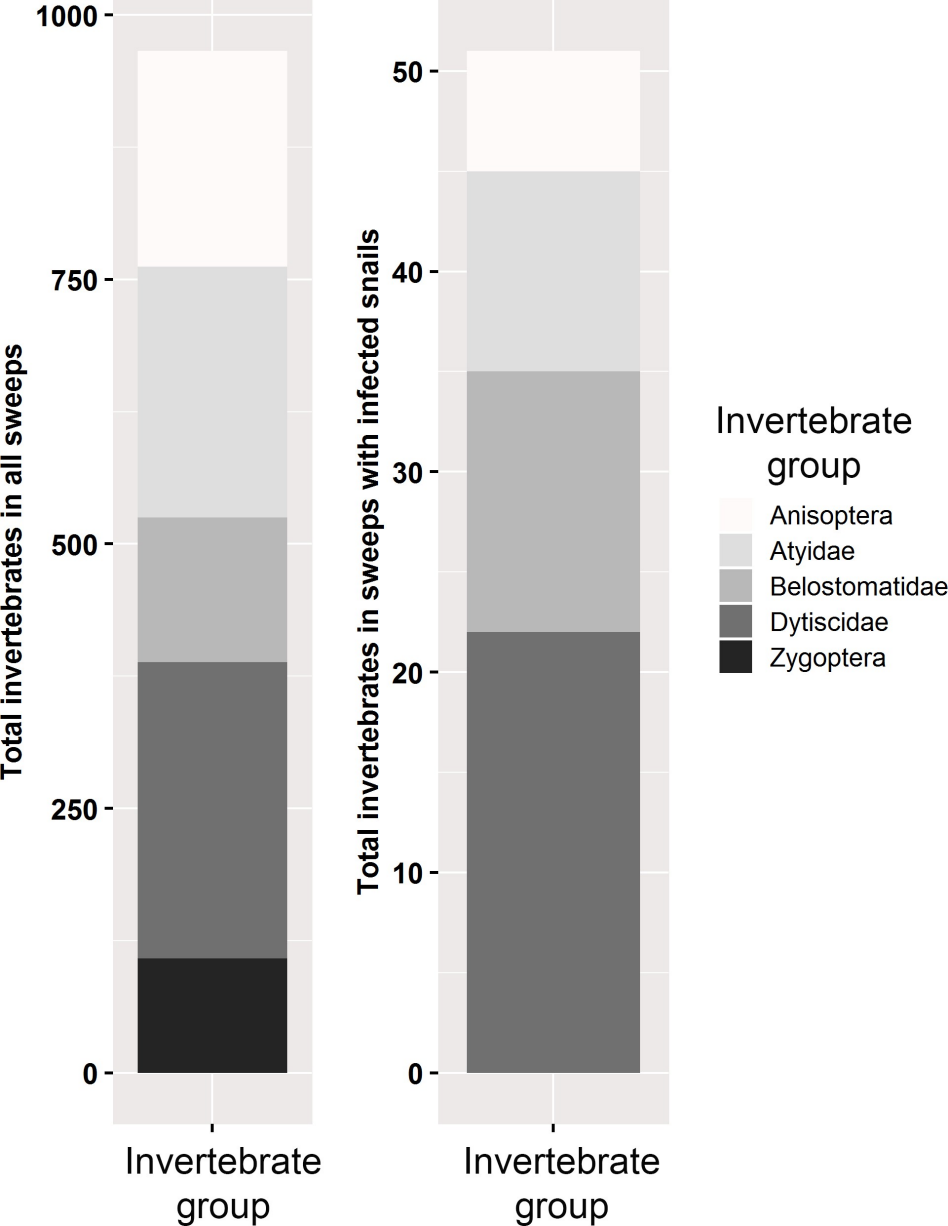
Abundance of invertebrate groups captured during 1-m aquatic net sweeps in the study (*n* = 540) or within sweeps having infected snails (*n* = 39).

We collected several taxa of aquatic macroinvertebrates (Fig 5), including (in order of abundance) larval predaceous diving beetles (Dytiscidae), small freshwater shrimp (Atyidae), larval dragonflies (Anisoptera), creeping water bugs (Naucoridae), and larval damselflies (Zygoptera). Of the macroinvertebrates at these sites, we were particularly interested in predaceous diving beetles (Dytiscidae), dragonflies (Anisoptera), water bugs (Naucoridae), and damselflies (Zygoptera), because laboratory foraging experiments have shown that all these invertebrate groups are predators of freshwater snails when snails are small [33]. By foraging on snails, many of these invertebrate groups can regulate snail populations [33] and suppress snail foraging [34]. We were interested in snail predation effects, and thus excluded the atyid freshwater shrimp from all analyses because they are grazers that might compete with freshwater snails for food, but do not depredate snails. Other snail predators, including large *Macrobrachium* spp. prawns were not quantified by our methods. Prawns and fish that prey on snails have previously been found in low abundance in our study region [44, 45].

A total of 967 aquatic macroinvertebrates were captured, and the number of macroinvertebrate predators per sweep was associated positively with the abundance of *Bulinus* (*χ*^*2*^ = 27.9, *df* = 1, *p* < 0.001; Fig 3C) and *B*. *pfeifferi* (*χ*^*2*^ = 17.2, *df* = 1, *p* < 0.001; Fig 3D) snails per sweep. A sweep-level model using each invertebrate taxa suggested that three taxonomic groups of predators (Belostomatidae, Zygoptera, and Dystiscidae) were significantly positively associated with snail abundance, which was consistent with the overall positive trend between total invertebrates and both *Bulinus* and *Biomphalaria* (S6 Table; S4 Fig). Although we did not have sufficient data on the abundance of each taxa within sweeps having infected snails (due to low snail infection) to model the influence of individual predator taxa on cercarial production, the percentage of predators from each group was consistent in both in the total sweeps and sweeps having infected snails (Fig 5). There was no strong association between snail abundance and macroinvertebrates at the site-level (access point) for either *Bulinus* spp. or *B*. *pfeifferi* (*p* > 0.05). Snail predators were associated positively with average *Bulinus* spp. shell diameter per site (*χ*^*2*^ = 7.7, *df* = 1, *p* = 0.005; Fig 4C), controlling for the effect of *Ceratophyllum* spp. mass (*χ*^*2*^ = 0.08, *df* = 1, *p* = 0.783), but this effect was not significant at the sweep-level (Fig 4B). Macroinvertebrate snail predators were negatively but marginally non-significantly associated with *Bulinus* spp. snail infection prevalence in a sweep (*χ*^*2*^ *=* 3.4, *df =* 1, *p =* 0.066; S1C Fig). Piecewise-path modeling also supported that invertebrate predators were positively associated with snail counts but did not significantly reduce snail prevalence (S2 and S3 Figs). In other words, in the presence of higher numbers of invertebrate predators, more snails were encountered on average per sweep, and larger snails tended to be encountered per site.

### Effects of aquatic vegetation and predators on cercarial abundance and snail infection

**Fig 6:**
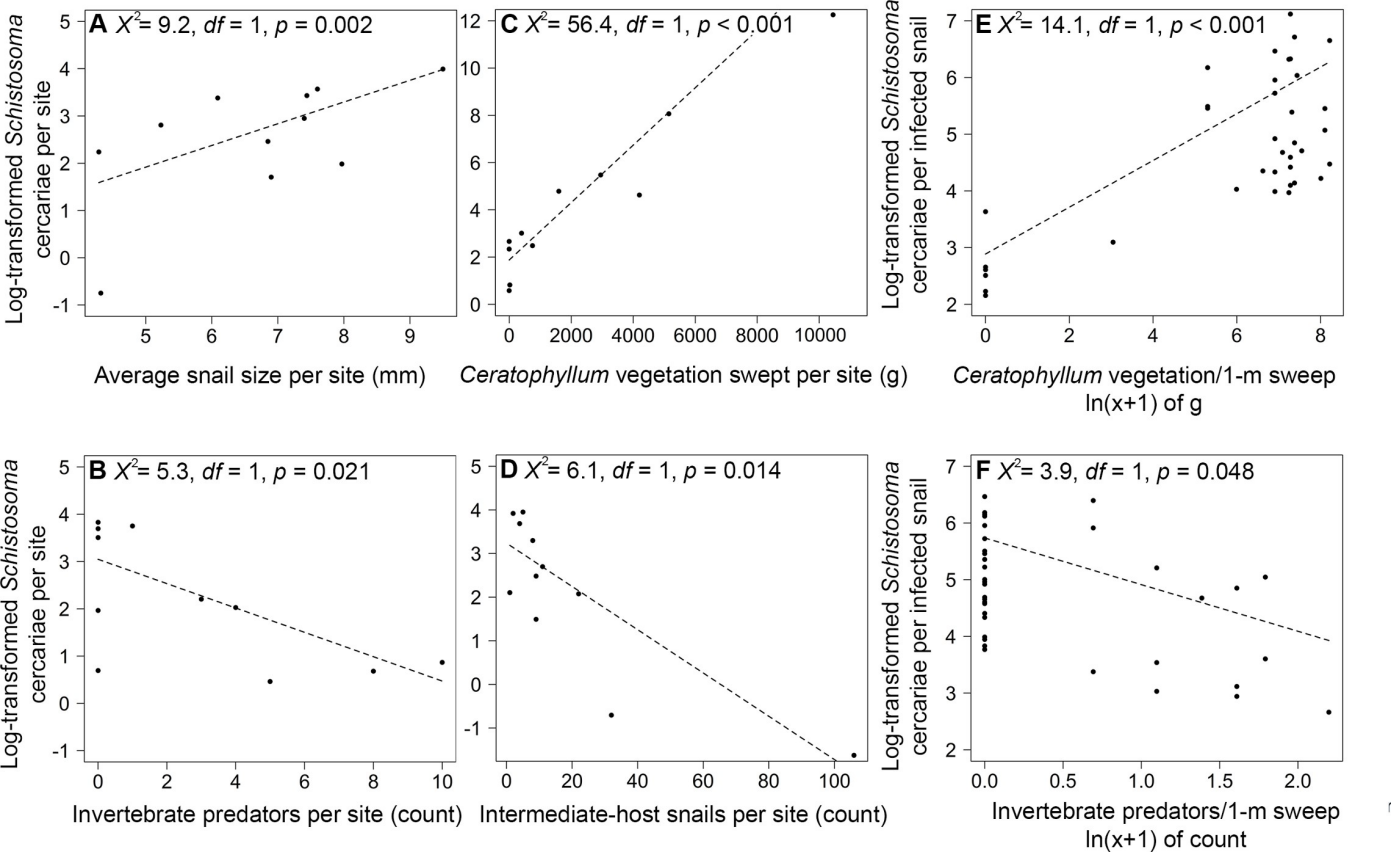
**Partial residual plots showing results from a negative binomial multiple regression model examining the effects of average snail size (A), invertebrate predator abundance (B), *Ceratophyllum* spp. mass (C), and snail abundance (D) on the sum of *Schistosoma* cercariae across sweeps per site (*n* = 11), and a separate negative binomial model examining the effects of *Ceratophyllum* spp. mass (E) and total invertebrate predator abundance (F) on *Schistosoma* cercariae per infected snail (*n* = 39; sweep nested within site as random effects).** In both models, only sweeps containing infected snails were included.

We found approximately 2.6% infection prevalence in snails (pooling all fork-tailed cercariae identified morphologically as most likely human and not avian schistosomes) for a total of 30 infected *Bulinus* spp. and 9 infected *B*. *pfeifferi* snails. Infected *Bulinus* spp. shed an average of 178 ± 68 (SE) cercariae with a range of 1 to 910, and *B*. *pfeifferi* shed an average of 258 ± 122 cercariae with maximum of 1,890. Infected *Bulinus* spp. had smaller average shell diameter (8.2 mm ± 0.46) than infected *B*. *pfeifferi* (9.9 mm ± 0.46). However, the number of cercariae shed per snail was not significantly related to shedding snail shell diameter (*χ*^*2*^ = 0.0, *df* = 1, *p* = 0.866), average snail shell diameter in the sweep (*χ*^*2*^ = 1.8, *df* = 1, *p* = 0.183), or snail abundance (*χ*^*2*^ = 0.1, *df* = 1, *p* = 0.715) after controlling for vegetation and macroinvertebrates (S7 Table). The sum of cercariae emitted from infected snails encountered across all sweeps at the site level (S8 Table) was positively associated with average snail shell diameter across the whole site (*χ*^*2*^ = 9.2, *df* = 1, *p* = 0.002; Fig 6A) and negatively associated with estimated snail abundance at the site level (count of all captured snails at a site; *χ*^*2*^ = 6.1, *df* = 1, *p =* 0.014; Fig 6D).

**Fig 7:**
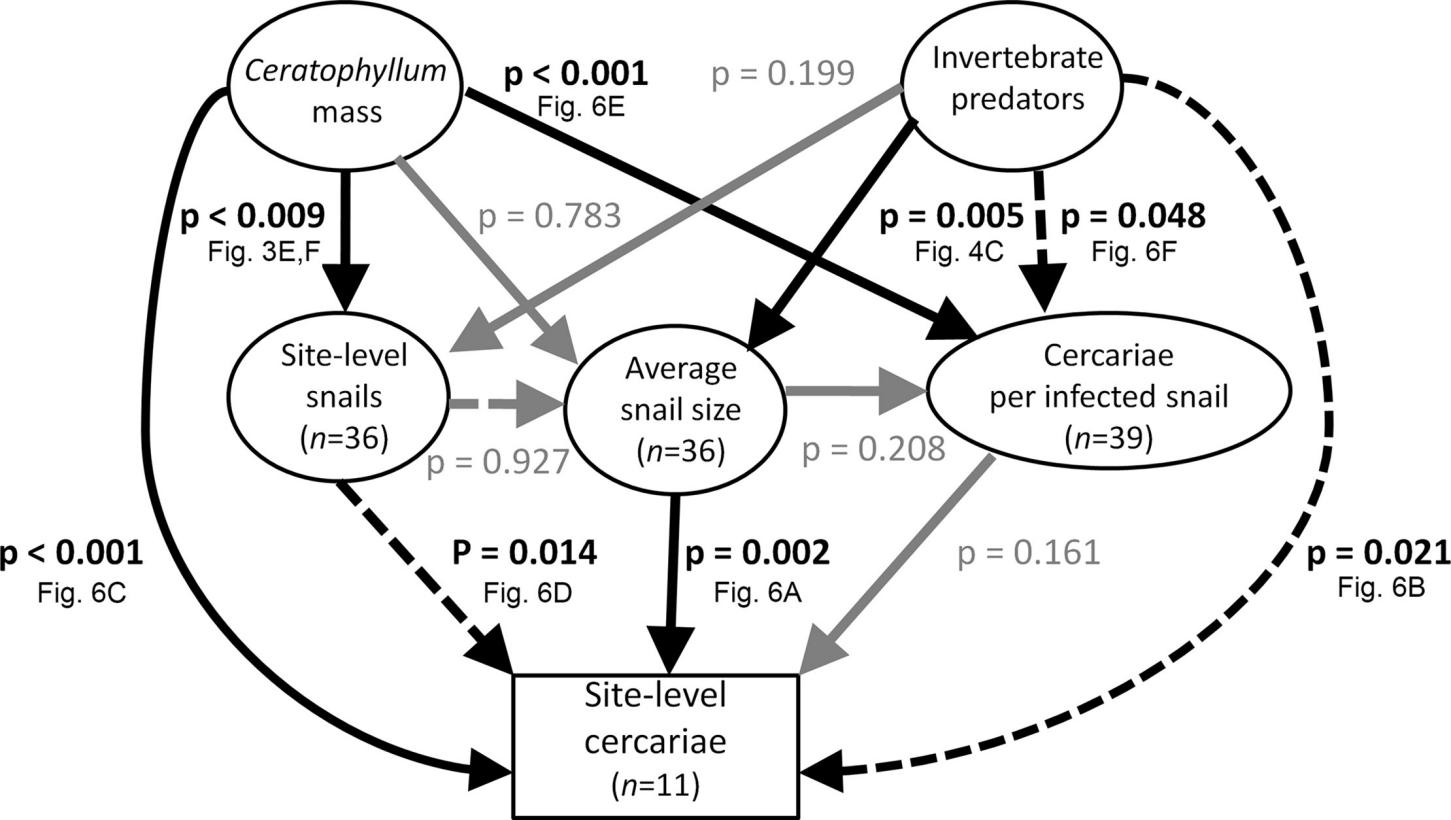
Summary of four individual regression analyses that examine the effects of abundance of aquatic vegetation (*Ceratophyllum* mass) and invertebrate snail predators on snail shell diameter, snail abundance, per capita cercarial release by infected snails, and potential cercarial production at a site. *P*-values next to each path represent the probability of the null hypothesis in each individual multiple regression model based on a log-likelihood ratio Chi-square test. Solid arrows are positive effects, dashed arrows are negative effects, and gray paths are non-significant paths. Analyses are hierarchical using all infected snails for cercariae per infected snail (*n* = 39, all sites for number of snails and average snail shell diameter (*n = 36*), and sites with infected snails for sum of cercariae released by snails encountered across all sweeps at a water access site (*n = 11*). We also conducted a true path model (S2 and S3 Figs) but utilized this hierarchical approach because of inconsistency in sample sizes across analyses that led to substantial loss of power using path analyses (see Methods); however, we display the regressions visually like a path analysis. We did not perform a multiple comparisons correction on the analyses because most included different datasets (albeit with some common data) and per the arguments against adjusting *p*-values for multiple comparisons by Gotelli and Ellison [46].

Of the 22 unique sweeps with infected snails, the number of cercariae released from infected snails increased significantly with the abundance of aquatic vegetation in the sweep (*χ*^*2*^ = 14.1, *df* = 1, *p* < 0.001; Fig 6E). Although infected snails occurred in a variety of aquatic vegetation (*Ceratophyllum* spp., *Potamogeton* spp., *Ludwigia* spp., and *Cyperus* spp.), they were found in only six sweeps lacking *Ceratophyllum* spp. Four of six sweeps with infected snails but no *Ceratophyllum* spp. were located in *Ludwigia* spp. (all *Bulinus* spp.), and one infected snail each was found on *Cyperus* spp. (*B*. *pfeifferi*) and dead *Typha* spp. stems (*Bulinus* spp.). Additionally, total *Ceratophyllum* spp. mass was associated positively with the sum of cercarial counts at the site level (*χ*^*2*^ = 56.4, *df* = 1, *p* < 0.001; Fig 6C), controlling for other factors (Fig 7). Piecewise path modeling also suggested positive total (direct and indirect) effects of *Ceratophyllum* spp. mass on potential cercariae released by encountered snails at a site by increasing either average snail abundance at a site, infection prevalence, or average per-capita cercarial release by infected snails (S2 and S3 Figs). In summary, controlling for shedding snail size, snail population, and predators, *Ceratophyllum* spp. mass was the strongest positive determinant of increased cercarial production per infected snail and per water-access point.

When controlling for shedding snail shell diameter (individual level), snail abundance, average snail shell diameter in a sweep, and *Ceratophyllum* spp. mass (from sweeps with infected snails) (Fig 6), the abundance of macroinvertebrate predators in a sweep was negatively associated with the per-capita cercarial release by infected snails (*χ*^*2*^ = 5.3, *df* = 1, *p* = 0.021; Fig 6B). When accounting for the same predictors across all sweeps having infected snails, invertebrate predators were negatively correlated with the total cercariae released from all snails encountered at the site-level (*χ*^*2*^ = 3.9, *df* = 1, *p* = 0.048; Fig 6F). Piecewise path models showed no significant correlation between predator counts and total cercariae released from snails encountered at a site. Importantly, *Ceratophyllum* effects on cercariae counts at the site-level were stronger than predator effects in all analyses (Figs 6, S2 and S3).

## Discussion

Our study demonstrated that submerged aquatic plants in the genus *Ceratophyllum*, which are common in other parts of Africa [18] and found on every continent where schistosomiasis is endemic, harbored over 70 percent of the infected snails and contained five and twenty-six times greater snail densities than emergent vegetation and open water, respectively. Despite *Ceratophyllum* spp. vegetation mass having no effect on average snail shell diameter, it was positively associated with per-capita cercarial release when compared to infected snails found in open water or reed-dominated habitats, likely because of increased periphyton availability on the high surface area of *Ceratophyllum* spp. and the associated improved snail body condition prior to and during parasite production [20]. These higher cercarial outputs could potentially increase human exposure risk for *S*. *mansoni* and *S*. *haematobium* at water access sites where *Ceratophyllum* spp. dominate, and where aquatic snail predators are rare. Indeed, where aquatic macroinvertebrate snail predators were present among *Ceratophyllum* spp. patches, infected snails exhibited lower per-capita cercarial production, suggesting potential reduction in human infection risk associated with non-lethal effects of small predators. Our results are consistent with a previously documented preference of *Bulinus* snails for *Ceratophyllum* spp. in West Africa [16, 18], but uniquely demonstrates that both vegetation and predators can affect the potential per-capita cercarial output by infected snails at water-access points. These findings suggest that ecological indices like plant and predator abundance might complement snail sampling for targeting public health interventions. Perhaps some of these habitat-host-parasite-predator interactions can explain why snail densities appear to be difficult to correlate with human prevalence [47–51] (but see[16]).

### Effects of aquatic vegetation and predators on snails

Submerged aquatic macrophytes, such as *Ceratophyllum* spp., might benefit snail populations by increasing periphyton, the food source for *Bulinus* spp. and *Biomphalaria pfeifferi* snails [52, 53]. There are several mechanisms by which macrophytes can enhance periphyton. Periphyton needs a surface on which to grow, and *Ceratophyllum* spp. provides high structural complexity compared to other submerged plants [53]. In the absence of submerged macrophytes, periphyton would typically have to grow on the sediment or objects that sink to the sediment. Given that light attenuates through the water column and photosynthetic organisms in the periphyton require light, periphyton is often restricted to shallow regions of waterbodies in the absence of macrophytes. However, because many submerged macrophytes, such as *Ceratophyllum* spp., are lightly rooted and float near the surface of the water where there is considerable light, the presence of macrophytes can provide a complex architecture and surface area upon which periphyton can attach and thus snails can feed [54]. Importantly, the relationship between *Ceratophyllum* spp. and snails might be mutualistic. Periphyton growing on *Ceratophyllum* intercepts light before it reaches the *Ceratophyllum* spp. plant tissues. Hence, by grazing on periphyton, the snails might serve as *Ceratophyllum’s* “cleaning symbiont”, allowing more light to reach the macrophyte that can increase its growth, lifespan, and potential dominance in aquatic habitats [54–56]. This, in turn, presumably further increases snail habitat, snail population growth, and potentially, the risk of schistosomiasis for people [18, 53].

In contrast to submerged macrophytes, emergent vegetation grows above the water column and thus intercepts light before it reaches the surface of the water. Consequently, the low water-column light associated with emergent vegetation might make it less suitable habitat for periphyton, and thus snails, than submerged vegetation. This is consistent with the observed low density of snails in emergent vegetation relative to submerged vegetation. Muddy river or lake bottom has less surface area for periphyton than any vegetation, consistent with this open water habitat having the lowest density of snails.

Consistent with past studies [57], we showed that macroinvertebrate predators are relatively dense in *Ceratophyllum* spp. Additionally, their abundance was positively correlated with snail abundance at large spatial scales, presumably because they have similar habitat requirements (for food or refuge) and/or are tracking their snail prey [58, 59]. At smaller spatial scales, predators can be correlated negatively with their prey if they have strong lethal effects [60, 61]. However, if snails evade predators by reaching a physical or size refuge, these significant relationships can break down [62]. In fact, we did not observe a significant negative correlation between macroinvertebrate predators and snail densities at the sweep level, suggesting that macroinvertebrate predators of snails might not be limiting snail populations. However, small macroinvertebrate predators still might change schistosomiasis risk by reducing snail infection prevalence, preferentially consuming infected snails, consuming cercariae, or exerting sublethal effects on snails by altering their behavior [31, 32]. Our study did not have sufficient captures of different invertebrate taxa to separately investigate their influence upon parasite production, and quantifying such variation might contribute to our understanding of mechanism or strength of the relationship between predators and parasite production.

### Effects of aquatic vegetation and predators on cercarial abundance and snail infection

In addition to our finding that *Ceratophyllum* spp. abundance is positively associated with snail densities, we also found that it is positively associated with per-capita cercarial release by infected snails encountered at a sweep or a site. Bottom-up effects, whereby snail food resources fuel snail growth and parasite production [20, 63, 64], could account for a significant increase in cercarial release from infected snails found in *Ceratophyllum* spp., compared to localities without this plant. As cercariae develop within infected snails, energy consumed by snails via grazing is diverted to parasite production [20].

*Ceratophyllum* spp. exhibit rapid growth from short-term nutrient pulses because of their high nutrient uptake [65], suggesting that future studies are needed to investigate how fertilizer inputs from agriculture adjacent to water access sites might enhance *Ceratophyllum* spp. growth and cercarial release by snails. In contrast, dense *Ceratophyllum* spp. patches might limit cercarial (and/or miracidial) movement by blocking water flow, which could either increase or decrease cercarial density at a site, depending on the balance between the plant’s trapping of locally released cercariae and its blocking of distantly produced cercariae from upstream.

The positive association between average snail size and infection prevalence is probably due in part to a positive age-intensity relationship, whereby larger snails are more likely to be older snails that have had longer exposure to miracidia over their lifespan than smaller snails [66]. Very low encounter and infection rates for *B*. *pfeifferi* suggest that our analyses of this species might be underpowered and future studies are needed to further clarify the drivers of *B*. *pfeifferi* infection dynamics. Given that *Ceratophyllum* spp. abundance is correlated with per-capita cercarial production as well as higher snail densities, this plant might be a predictor of human schistosomiasis risk [16], and this warrants further study.

We identified a negative but complex relationship between the abundance of macroinvertebrate snail predators and the total number of cercariae released from snails encountered at a site, and this observation might help inform schistosomiasis control. Macroinvertebrate predators can induce several anti-predator behaviors in *Bulinus* and *Biomphalaria* spp. snails, such as retreating inside their shells, leaving the water, or moving below substrates [32]. All of these sublethal effects are likely to reduce snail grazing, which may have contributed to the observed negative relationship between macroinvertebrate predators and cercarial production among the infected snails we encountered (Fig 6B and 6F). Previous studies on a crustacean snail predator, *Macrobrachium* spp., suggested that the predator preferentially attacks and consumes infected snails. In this way, predators might reduce snail infection prevalence by lethal effects [32]. However, the smaller macroinvertebrate snail predators that we sampled here were positively associated with average snail shell diameter at a site, probably because they consumed only the smallest snails while the larger snails had a size refuge [67–69]. Such selective predation of smaller snails may also explain our weak evidence for any impact of small macroinvertebrate predators on snail infection prevalence, because smaller, younger snails have less parasite exposure than larger, older snails, and are less likely to have reached the patent shedding stage of infection [66]. However, small macroinvertebrates can also act as decoys to miracidia seeking to infect snails [70]. Other studies have suggested that non-lethal trait-mediated effects of predators can be stronger than density-mediated effects, particularly within aquatic systems [71]. Indeed, our data support the hypothesis that snails might limit their foraging in the presence of predator cues, which can reduce their food intake and growth [29], and consequently their cercarial production [66]. The balance of these myriad lethal and non-lethal effects of small macroinvertebrate predators on snails and their trematode infections requires further study. Our sampling methods also could not assess the impact of larger fish and invertebrates (like prawns) that may consume snails or *Ceratophyllum* spp, but are generally at low abundance in this region [45].

The fact that we did not find any association between individual snail shell diameter and snail shedding rate in the field may be due to several factors. First, the rate of parasite penetration into snails may be non-monotonic, wherein small snails produce fewer scent molecules that attract the parasite, and larger snails can become more resistant to their penetration [72]. Second, snails could become infected at a large size and continue to use some energy for reproduction, rather than parasite production, increasing numbers of smaller snails that both compete for resources with infected snails and can themselves shed fewer parasites [66, 73, 74] (see Fig 6A). Third, variation in schistosome egg deposition through human excreta at water access sites is a factor difficult to control for in field studies that is likely to influence the number of miracidia reaching snails, and therefore the prevalence and cercarial production in snails [72]. Our study was strictly correlational and further experiments will be needed to distinguish among competing hypotheses.

We suspect that the considerable variation among infected snails in the amount of cercariae that they release is the reason why snail density near infected snails was a less significant predictor of potential cercarial abundance than aquatic vegetation or predators. Overall, Civitello et al. (2018) showed that resource-dependent production of parasites by snails drove the majority of variation in snail infection dynamics, whereas the density of *Biomphalaria glabrata* snails in outdoor mesocosms was a poor predictor of total cercarial release by infected snails. In that study, as snail density increased, competition for resources led infected snails to produce very few cercariae, resulting in a very weak correlation between snail and cercarial densities. We too detected a negative correlation between snail densities and cercarial production (Fig 6D), consistent with the hypothesis that snail resources might become limited in nature and that negative density dependence might be regulating snail populations at some of our sites. However, further research will be needed to demonstrate this definitively using larger sample sizes and with sampling across time. Overall, when averaged across all sweeps, path analyses suggested that aquatic vegetation can increase cercariae per site by increasing snail abundance, snail infection prevalence, and/or parasite production per infected snail (S2 and S3 Figs). Similarly, our hierarchical regression approach (using only sweeps having infected snails as the replicate) confirmed a strong positive effect of *Ceratophyllum* spp. mass on per-capita parasite production and total infected snail abundance. This latter analysis suggested that, despite a positive association between aquatic predator and snail abundances, predators near snail food resources might still lower their per-capita parasite production (Fig 7). Snail or cercarial counts may offer better prediction of disease transmission to humans than found in previous studies [19] when aquatic snail habitat quantity at water access sites is taken into account [16]. Future studies are needed to differentiate potential impacts of aquatic plants and macroinvertebrates on human schistosomes from those on non-human schistosome, hybrid and other cryptic species of forked-tailed cercariae emitted from the same snails in this study region. Moreover, the historically weak correlation between snail abundance and human schistosomiasis prevalence [13, 14], and mismatch between snail and cercarial abundances [19], might be influenced by the considerable heterogeneity we observed in cercarial production among infected snails.

There are a number of caveats and limitations to our study. We did not use molecular techniques required to differentiate human schistosome cercariae from non-human schistosomes or other cryptic trematode cercariae (such as bird or wildlife parasites) emitted from the same snail species. However, we think the impact of potential misclassification of fork tailed cercariae on our study findings was low because *i*) the most common non-human schistosome, *S*. *bovis*, was avoided by shedding later in the day than the peak period for this species, *ii)* other fork tailed cercariae were screened using morphology of their eyespots, tails, and finfolds, and *iii*) data collected (personal communication) by our co-authors at the same study sites had suggested to us that non-human or avian schistosome cercariae in our study region are uncommon (approximately 15% of fork-tailed cercariae) relative to human schistosomes, as has been shown in other highly endemic schistosomiasis regions [75, 76]. Additionally, while we cannot distinguish *S*. *bovis* from *S*. *haematobium* that can be shed by the same *Bulinus* spp., co-infection within a single snail is rare [77]. Thus, while we acknowledge a complex interplay between trematodes that might have occurred within the examined snails, we think that the ecological effects we investigated–aquatic macrophyte and non-lethal predator effects on cercarial production–were likely conserved across any closely related but undifferentiated, trematode species emitted from these snails, which were likely rare. Additionally, we focused sampling in the summer wet season when human transmission is high; patterns could be different in the dry season.

We also did not perform cercariometry at these sites to confirm whether the sum of cercariae emitted from encountered snails correlated with other standardized measures of human risk at each site. This could have implications for our findings because cercariae can travel considerable distance, especially with the assistance of water flow. By bringing snails into the laboratory and shedding them, we were isolating parasite production from just the snails we captured in our sweeps; by doing so we aimed to avoid confounding effects of parasites released at other locations that drifted or swam into the associated sampling point. Various factors can affect cercarial survival and movement and thus it is unclear what the likelihood is that cercariae emitted from our collected snails would have posed risk to humans in the field, had they not been collected and taken to the laboratory for shedding. Additional environmental factors, such as wind and stream flow, could bring in cercariae from infected snails residing some distance from the focal water access site. It is for this reason that we chose not to use eDNA-based approaches to quantify schistosome cercariae in the water. We were interested in examining the effects of vegetation and predators on local cercarial production, which is why we shed isolated snails. In contrast, eDNA-based approaches would confound locally produced cercariae with those that swim or drift in from elsewhere. Another reason we avoided eDNA-based approaches is because there are no molecular methods to distinguish miracidial from cercarial densities of the same schistosome species, and thus, unlike our shedding approach, eDNA approaches would confound the two life stages.

### Management applications

Our findings that aquatic vegetation and insect predators influence both snail abundance and per-capita release of cercariae at water access sites offers important insights for understanding human risk of schistosomiasis. Cercarial density and abundance in the water are primary drivers of human risk [78]. Per-capita cercarial release by infected snails is likely dependent upon snail resources [20], and consistent with this mechanism, we found increased per capita cercarial release in snails collected at sites with high *Ceratophyllum* spp. mass and/or low predator abundance. While we did not measure agrochemicals in the water at these sites, previous work suggests that fertilizers are abundant in the Senegal River Basin and probably fuel macrophyte (e.g. *Ceratophyllum* spp.) growth; insecticides have also been detected in the water and are highly lethal to macroinvertebrate predators of snails like the ones we encountered [26]. These observations suggest that agrochemicals and nutrient runoff from agriculture could indirectly influence cercarial abundances or snail infection, and therefore schistosomiasis risk, through effects on macrophytes and snail predators. Indeed, Halstead et al. (2018) showed that both fertilizers and the insecticide chlorpyrifos increased snail abundance through bottom-up and top-down effects in mesocosms, and the results of our study are consistent with, but not confirmatory of, the idea that this mechanism might also act under field conditions. If these results are indeed confirmed in larger studies, it compels us to consider potential management options to reduce nutrient runoff such as installing buffer zones, whereby crops are not planted close to the land–water interface, or avoiding the most risky options among fertilizers and insecticides that might influence schistosome shedding by snails in the field. Importantly, while previous work documented that removing *Ceratophyllum* spp. can reduce infected snail counts [18], our findings suggest that it also may simultaneously reduce per-capita cercarial release by infected snails, especially if the remaining snails compete more strongly for habitat and limited energy sources [20], further lowering human re-infection risk following chemotherapy. However consistent with laboratory and mesocosm findings, there is a strong need to verify our correlational research using scaled up experimental studies that manipulate snail resources or agrochemical application rates across large spatial extents, while tracking changes to parasite counts in snails and in the water, and ultimately human infection or re-infection.

## Supporting information

S1 Fig Partial residual plots showing results from a binomial multiple regression model examining the effects of average snail size (A), *Bulinus* snail abundance (B), and invertebrate predator abundance (B), on sweep-level *Bulinus* infection prevalence, controlling for the non-significant effect of *Ceratophyllum* spp. mass (*p* = 0.884).
(TIF)

S2 Fig Piecewise-path analysis plot for all schistosome intermediate host snails with significant (*p* < 0.05) pathways as black arrows, marginally non-significant (*p* < 0.10) pathways as gray arrows, and standardized coefficients provided in boxes on each pathway. Path analysis results using site-level average values agreed with our hierarchical regression approach, indicating that average *Ceratophyllum* mass increased average cercariae per infected snail and average snail abundance, which in turn increased cercariae per site. Effect of invertebrate predators on cercariae per site that were significant in the sweep-level hierarchical regression analysis, were not significant in the site-level path model, likely due to spatial heterogeneity of predator counts within *Ceratophyllum* that leads to a loss of statistical power when averaging values for all sweeps at the site level.
(TIF)

S3 Fig Piecewise-path analysis plot using data from only *Bulinus* spp. snails with significant (*p* < 0.05) pathways as black arrows, marginally non-significant (*p* < 0.10) pathways as gray arrows, and standardized coefficients provided in boxes on each pathway. Path analysis results using site-level average values agreed with our hierarchical regression approach, indicating that average *Ceratophyllum* mass increased average cercariae per infected snail and average snail abundance, which in turn increased cercariae per site. Effect of invertebrate predators on cercariae per site that were significant in the sweep-level hierarchical regression analysis were not significant in the site-level path analysis than they were, likely due to spatial heterogeneity of predator counts within *Ceratophyllum* that leads to a loss of statistical power when averaging values for all sweeps at the site level.
(TIF)

S4 Fig Partial residual plots based on a final negative binomial multiple regression after model selection using *Bulinus* spp. counts at the sweep-level as the dependent response variable and *Ceratophyllum* spp. mass and invertebrate predator abundance per taxa as independent variables. (TIF)

S1 Table List of predictor and response variables used in the starting full piecewise-path model, prior to model selection, where values of 1 indicated whether a path was included in the starting model. The starting model was based on *a priori* pathways and pathways recommended by tests of d-separation. Model selection was conducted by individually dropping non-significant pathways and comparing nested models by AIC. All variables were summarized at the site-level and snail and predator counts were log-transformed prior to averaging. We performed a path analysis for all schistosome snails (*Bulinus* and *Biomphalaria* combined dataset) and a separate model using data for *Bulinus* spp. snails only, with significant paths after model selection shown in S2 and S3 Figs.
(DOCX)

S2 Table Model selection by Akaike's Information Criteria for snail abundance among vegetation types. (DOCX)

S3 Table Model selection by Akaike's Information Criteria for sweep-level snail abundance. (DOCX)

S4 Table Model selection by Akaike's Information Criteria for site-level snail abundance. (DOCX)

S5 Table Model selection by Akaike's Information Criteria for average *Bulinus* spp. shell length. (DOCX)

S6 Table Model selection by Akaike's Information Criteria for *Bulinus* spp. abundance at the sweep-level using taxa-level invertebrate predator counts. (DOCX)

S7 Table Model selection by Akaike's Information Criteria for cercarial abundance per infected snail. (DOCX)

S8 Table Model selection by Akaike's Information Criteria for site-level cercarial abundance. (DOCX)
