# Supplementary figures and images for: Aquatic macrophytes and macroinvertebrate predators affect densities of snail hosts and local production of schistosome cercariae that cause human schistosomiasis
Source: PLoS Negl Trop Dis. 2020 Jul 6;14(7):e0008417. doi: 10.1371/journal.pntd.0008417 (PMC7365472; doi:10.1371/journal.pntd.0008417)

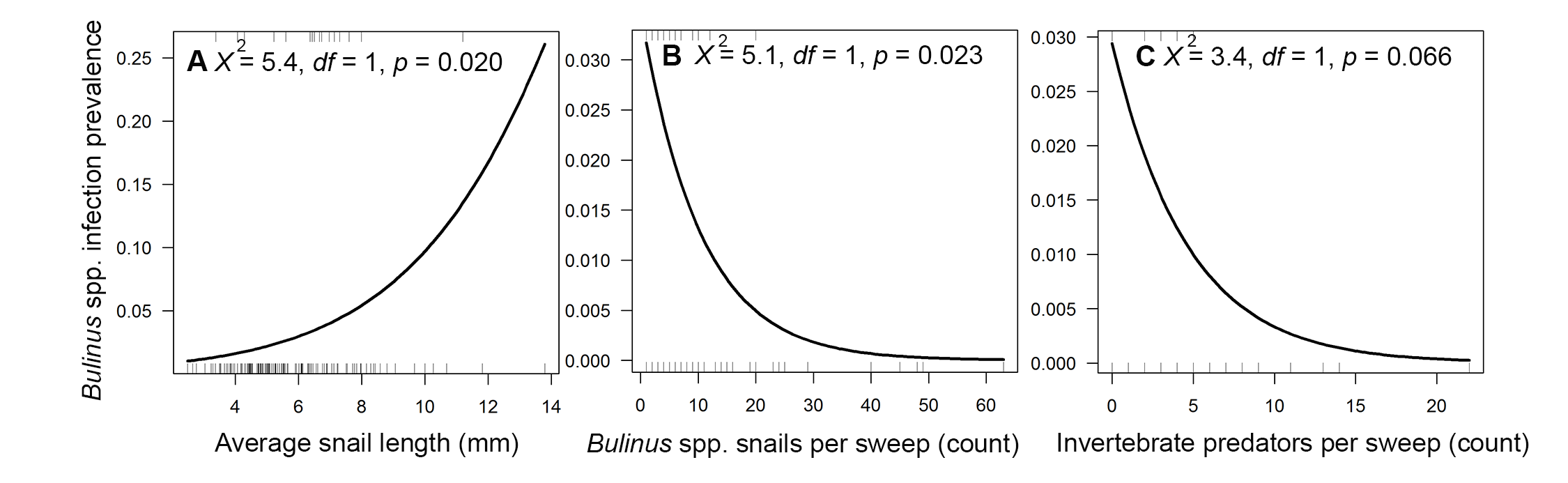

Supplement: S1 Fig — Partial residual plots showing results from a binomial multiple regression model examining the effects of average snail size (A), Bulinus snail abundance (B), and invertebrate predator abundance (B), on sweep-level Bulinus infection prevalence, controlling for the non-significant effect of Ceratophyllum spp. mass (p = 0.884). (TIF) [file pntd.0008417.s001.tif]

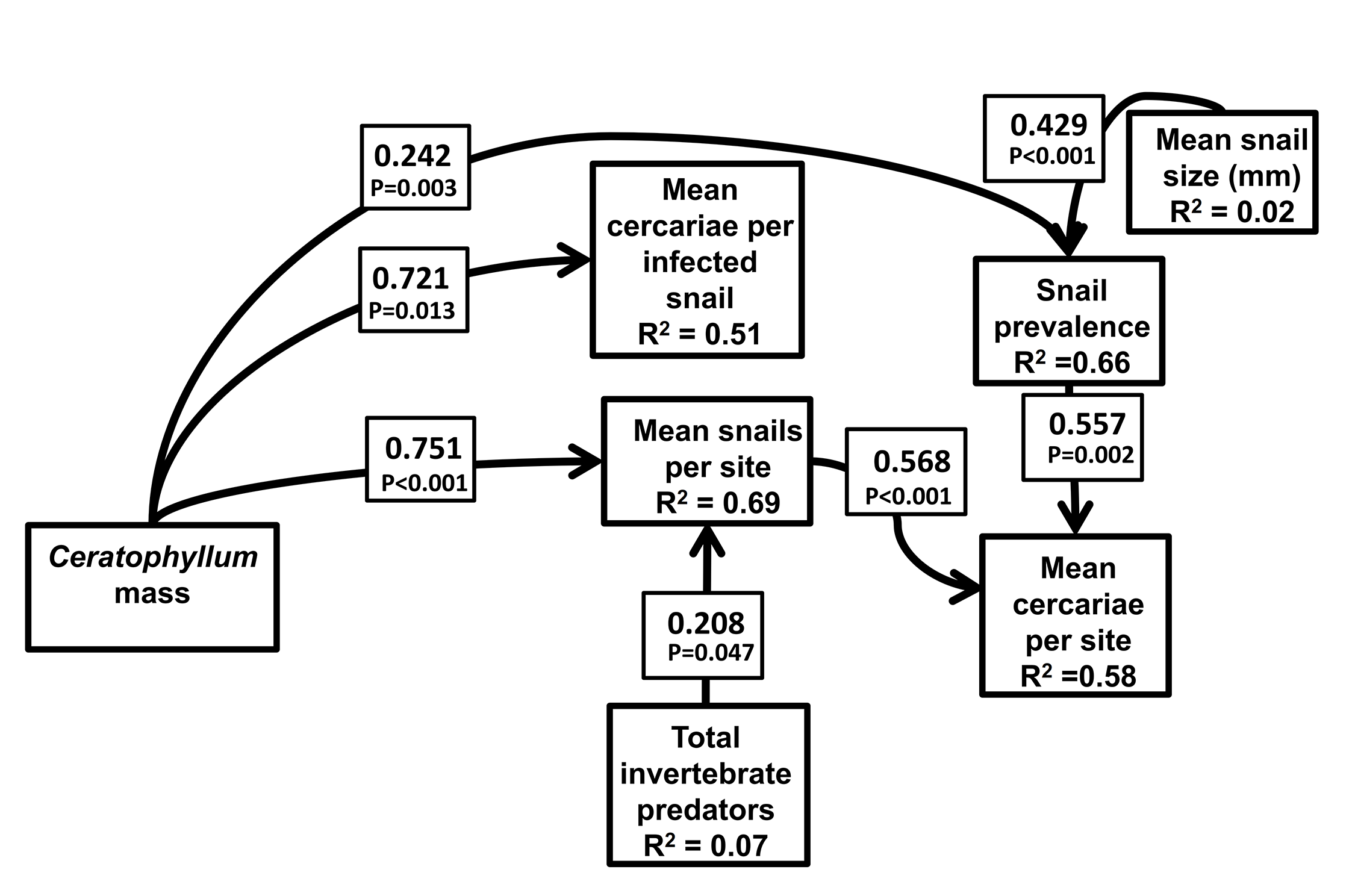

Supplement: S2 Fig — Path analysis results using site-level average values agreed with our hierarchical regression approach, indicating that average Ceratophyllum mass increased average cercariae per infected snail and average snail abundance, which in turn increased cercariae per site. Effect of invertebrate predators on cercariae per site that were significant in the sweep-level hierarchical regression analysis, were not significant in the site-level path model, likely due to spatial heterogeneity of predator counts within Ceratophyllum that leads to a loss of statistical power when averaging values for all sweeps at the site level. (TIF) [file pntd.0008417.s002.tif]

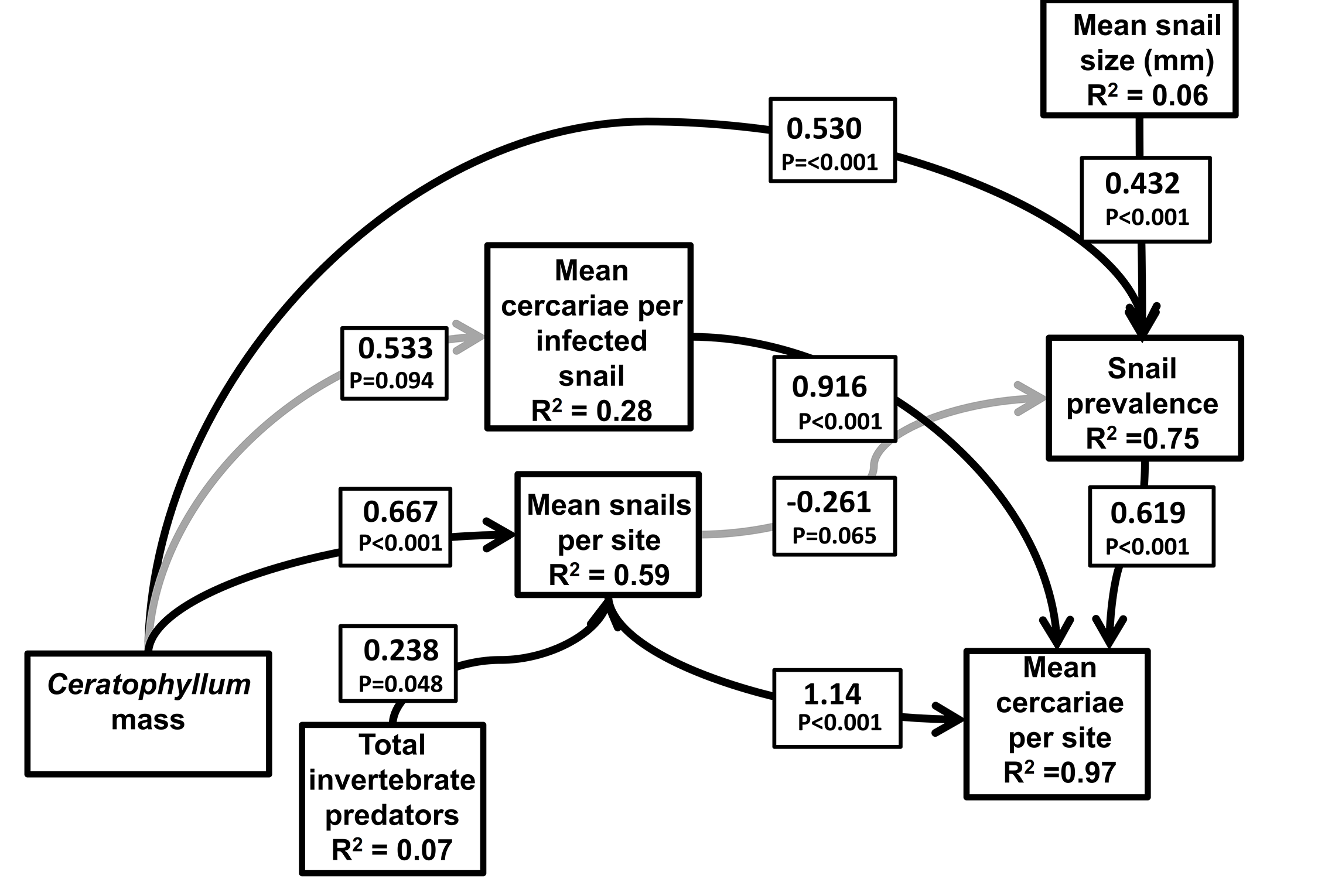

Supplement: S3 Fig — Path analysis results using site-level average values agreed with our hierarchical regression approach, indicating that average Ceratophyllum mass increased average cercariae per infected snail and average snail abundance, which in turn increased cercariae per site. Effect of invertebrate predators on cercariae per site that were significant in the sweep-level hierarchical regression analysis were not significant in the site-level path analysis than they were, likely due to spatial heterogeneity of predator counts within Ceratophyllum that leads to a loss of statistical power when averaging values for all sweeps at the site level. (TIF) [file pntd.0008417.s003.tif]

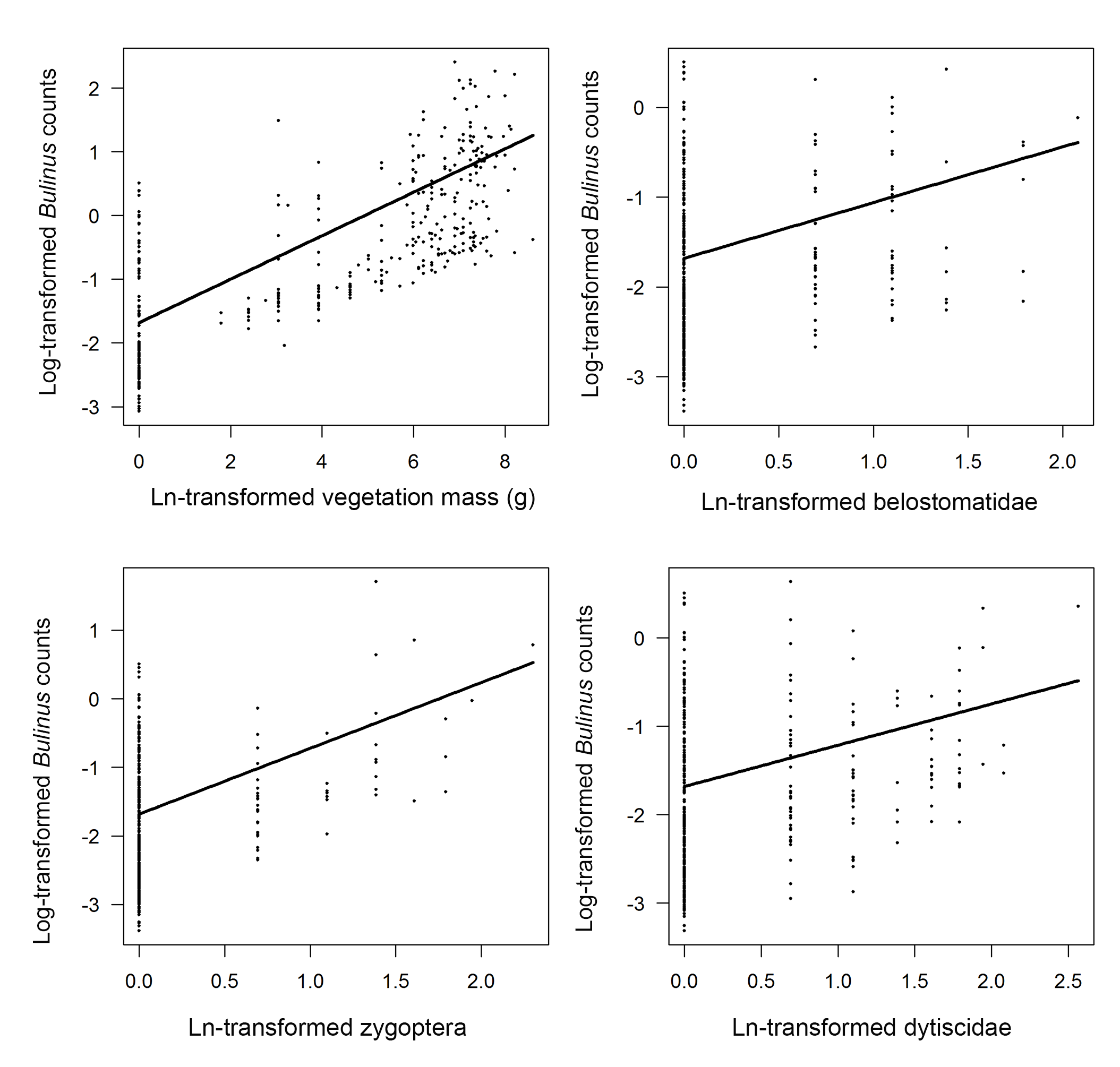

Supplement: S4 Fig — (TIF) [file pntd.0008417.s004.tif]
