# Supplementary material for: Aquatic macrophytes and macroinvertebrate predators affect densities of snail hosts and local production of schistosome cercariae that cause human schistosomiasis
Source: PLoS Negl Trop Dis. 2020 Jul 6;14(7):e0008417. doi: 10.1371/journal.pntd.0008417 (PMC7365472; doi:10.1371/journal.pntd.0008417)
Supplement: S1 Table — The starting model was based on a priori pathways and pathways recommended by tests of d-separation. Model selection was conducted by individually dropping non-significant pathways and comparing nested models by AIC. All variables were summarized at the site-level and snail and predator counts were log-transformed prior to averaging. We performed a path analysis for all schistosome snails (Bulinus and Biomphalaria combined dataset) and a separate model using data for Bulinus spp. snails only, with significant paths after model selection shown in S2 and S3 Figs. (DOCX) [file pntd.0008417.s005.docx]

**Table S1.** List of predictor and response variables used in the starting full piecewise-path model, prior to model selection, where values of 1 indicated whether a path was included in the starting model. The starting model was based on *a priori* pathways and pathways recommended by tests of d-separation. Model selection was conducted by individually dropping non-significant pathways and comparing nested models by AIC. All variables were summarized at the site-level and snail and predator counts were log-transformed prior to averaging. We performed a path analysis for all schistosome snails (*Bulinus* and *Biomphalaria* combined dataset) and a separate model using data for *Bulinus* spp. snails only, with significant paths after model selection shown in Figs. S2-S3.

| Paths from(row)/to(column): | *Ceratophyllum* mass | Mean log(schistosomes-shed/snail (conditional on snail is positive)) | | Mean snail size (mm) | Snail prevalence | | Mean log (snail count per sweep) | | Mean log(cercariae per sweep) | | Mean log(Total invertebrate predators per sweep) | |
| --- | --- | --- | --- | --- | --- | --- | --- | --- | --- | --- | --- | --- |
| *Ceratophyllum* mass |  | | 1 | 1 | | 1 | | 1 | | 1 | | 1 |
| Mean log (schistosomes-shed/snail (conditional on snail is positive)) |  | |  |  | |  | |  | | 1 | |  |
| Mean snail size (mm) |  | | 1 |  | | 1 | |  | | 1 | |  |
| Mean log (snail count per sweep) |  | |  | 1 | | 1 | |  | |  | |  |
| Mean log(cercariae per sweep) |  | |  |  | |  | |  | |  | |  |
| Mean log(Total invertebrate predators per sweep) |  | | 1 | 1 | | 1 | | 1 | | 1 | |  |
| Snail prevalence |  | |  |  | |  | |  | | 1 | |  |
