# Supplementary material for: Aquatic macrophytes and macroinvertebrate predators affect densities of snail hosts and local production of schistosome cercariae that cause human schistosomiasis
Source: PLoS Negl Trop Dis. 2020 Jul 6;14(7):e0008417. doi: 10.1371/journal.pntd.0008417 (PMC7365472; doi:10.1371/journal.pntd.0008417)
Supplement: S2 Table — (DOCX) [file pntd.0008417.s006.docx]

| **Table S2.** Model selection by Akaike's Information Criteria for snail abundance among vegetation types. | | | | | | |
| --- | --- | --- | --- | --- | --- | --- |
| Species | Single-term deletions | Df | AIC | ΔAIC | LRT | *p*-value |
| *Bulinus* spp. | None |  | 1538.9 |  |  |  |
| *Bulinus* spp. | Vegetation type | 2 | 1610.2 | 71.3 | 75.3 | <0.001 |
|  |  |  |  |  |  |  |
| *B. pfeifferi* | None |  | 624.6 |  |  |  |
| *B. pfeifferi* | Vegetation type | 2 | 643.8 | 19.2 | 23.2 | <0.001 |
