# Supplementary material for: Aquatic macrophytes and macroinvertebrate predators affect densities of snail hosts and local production of schistosome cercariae that cause human schistosomiasis
Source: PLoS Negl Trop Dis. 2020 Jul 6;14(7):e0008417. doi: 10.1371/journal.pntd.0008417 (PMC7365472; doi:10.1371/journal.pntd.0008417)
Supplement: S3 Table — (DOCX) [file pntd.0008417.s007.docx]

| **Table S3.** Model selection by Akaike's Information Criteria for sweep-level snail abundance. | | | | | | |
| --- | --- | --- | --- | --- | --- | --- |
| Species | Single-term deletions | Df | AIC | ΔAIC | LRT | *p*-value |
| *Bulinus* spp. | None |  | 1526.1 |  |  |  |
| *Bulinus* spp. | Predator abundance | 1 | 1553.9 | 27.8 | 29.8 | <0.001 |
| *Bulinus* spp. | *Ceratophyllum* spp. mass | 1 | 1588.7 | 62.6 | 64.6 | <0.001 |
|  |  |  |  |  |  |  |
| *B. pfeifferi* | None |  | 626.6 |  |  |  |
| *B. pfeifferi* | *Ceratophyllum* spp. mass | 1 | 632.6 | 6.0 | 8.0 | 0.005 |
| *B. pfeifferi* | Predator abundance | 1 | 641.2 | 14.6 | 16.6 | <0.001 |
