# Supplementary material for: Aquatic macrophytes and macroinvertebrate predators affect densities of snail hosts and local production of schistosome cercariae that cause human schistosomiasis
Source: PLoS Negl Trop Dis. 2020 Jul 6;14(7):e0008417. doi: 10.1371/journal.pntd.0008417 (PMC7365472; doi:10.1371/journal.pntd.0008417)
Supplement: S4 Table — (DOCX) [file pntd.0008417.s008.docx]

| **Table S4.** Model selection by Akaike's Information Criteria for site-level snail abundance. | | | | | | |
| --- | --- | --- | --- | --- | --- | --- |
| Species | Single-term deletions | Df | AIC | ΔAIC | LRT | *p*-value |
| *Bulinus* spp. | None |  | 313.9 |  |  |  |
| *Bulinus* spp. | Predator abundance | 1 | 314.0 | 0.1 | 2.1 | 0.148 |
| *Bulinus* spp. | *Ceratophyllum* spp. mass | 1 | 320.4 | 6.5 | 8.5 | 0.004 |
|  |  |  |  |  |  |  |
| *B. pfeifferi* | Predator abundance |  | 172.1 | 1.9 | 0.1 | 0.789 |
| *B. pfeifferi* | None | 1 | 174.0 |  |  |  |
| *B. pfeifferi* | *Ceratophyllum* spp. mass | 1 | 189.0 | 15.0 | 17.0 | <0.001 |
