# Supplementary material for: Aquatic macrophytes and macroinvertebrate predators affect densities of snail hosts and local production of schistosome cercariae that cause human schistosomiasis
Source: PLoS Negl Trop Dis. 2020 Jul 6;14(7):e0008417. doi: 10.1371/journal.pntd.0008417 (PMC7365472; doi:10.1371/journal.pntd.0008417)
Supplement: S5 Table — (DOCX) [file pntd.0008417.s009.docx]

| **Table S5.** Model selection by Akaike's Information Criteria for average *Bulinus* spp. shell length. | | | | | | |
| --- | --- | --- | --- | --- | --- | --- |
|  | Single-term deletions | Df | AIC | ΔAIC | LRT | *p*-value |
|  | None |  | 160.5 |  |  |  |
|  | Predator abundance | 1 | 164.8 | 4.3 | 6.3 | 0.121 |
|  | Snail abundance | 1 | 158.5 | 2.0 | 0.0 | 0.922 |
|  | *Ceratophyllum* spp. mass | 1 | 158.5 | 2.0 | 0.1 | 0.771 |
