# Supplementary material for: Aquatic macrophytes and macroinvertebrate predators affect densities of snail hosts and local production of schistosome cercariae that cause human schistosomiasis
Source: PLoS Negl Trop Dis. 2020 Jul 6;14(7):e0008417. doi: 10.1371/journal.pntd.0008417 (PMC7365472; doi:10.1371/journal.pntd.0008417)
Supplement: S6 Table — (DOCX) [file pntd.0008417.s010.docx]

| **Table S6.** Model selection by Akaike's Information Criteria for *Bulinus* spp. abundance at the sweep-level using taxa-level invertebrate predator counts. | | | | | |
| --- | --- | --- | --- | --- | --- |
| Single-term deletions | Df | AIC | ΔAIC | LRT | *p*-value |
| None | 1 | 1532.5 |  |  |  |
| Anisoptera | 1 | 1533.9 | 1.5 | 1.5 | 0.150 |
