# Supplementary material for: Aquatic macrophytes and macroinvertebrate predators affect densities of snail hosts and local production of schistosome cercariae that cause human schistosomiasis
Source: PLoS Negl Trop Dis. 2020 Jul 6;14(7):e0008417. doi: 10.1371/journal.pntd.0008417 (PMC7365472; doi:10.1371/journal.pntd.0008417)
Supplement: S7 Table — (DOCX) [file pntd.0008417.s011.docx]

| **Table S7.** Model selection by Akaike's Information Criteria for cercarial abundance per infected snail. | | | | | |
| --- | --- | --- | --- | --- | --- |
| Single-term deletions | Df | AIC | ΔAIC | LRT | *p*-value |
| Shedding snail mass | 1 | 445.0 |  |  |  |
| Snail abundance | 1 | 445.4 | 0.3 | 0.3 | 0.557 |
| Invertebrate predators | 1 | 446.2 | 1.1 | 1.1 | 0.289 |
| None |  | 447.0 | 2.0 | 0.0 | 0.982 |
| Average snail size | 1 | 447.4 | 2.4 | 2.4 | 0.122 |
| *Ceratophyllum* spp. mass | 1 | 449.1 | 4.1 | 4.1 | 0.043 |
