# Supplementary material for: Aquatic macrophytes and macroinvertebrate predators affect densities of snail hosts and local production of schistosome cercariae that cause human schistosomiasis
Source: PLoS Negl Trop Dis. 2020 Jul 6;14(7):e0008417. doi: 10.1371/journal.pntd.0008417 (PMC7365472; doi:10.1371/journal.pntd.0008417)
Supplement: S8 Table — (DOCX) [file pntd.0008417.s012.docx]

| **Table S8.** Model selection by Akaike's Information Criteria for site-level cercarial abundance. | | | | | |
| --- | --- | --- | --- | --- | --- |
| Single-term deletions | Df | AIC | ΔAIC | LRT | *p*-value |
| None |  | 110.8 |  |  |  |
| Cercariae per infect. snail | 1 | 111.3 | 0.5 | 2.5 | 0.117 |
| Snail abundance | 1 | 113.5 | 2.7 | 4.7 | 0.030 |
| Average snail size | 1 | 118.7 | 7.9 | 9.9 | 0.002 |
| *Ceratophyllum* spp. mass | 1 | 128.2 | 17.4 | 19.4 | <0.001 |
| Invertebrate predators | 1 | 113.8 | 3.0 | 5.0 | 0.026 |
| Snail infection prevalence | 1 | 109.7 | 1.1 | 0.8 | 0.358 |
